# *meso*-5,10,15,20-Tetra­kis(4-hy­droxy-3-meth­oxy­phen­yl)porphyrin propionic acid monosolvate

**DOI:** 10.1107/S1600536812036495

**Published:** 2012-08-25

**Authors:** Agnieszka Leonarska, Maciej Zubko, Piotr Kuś, Joachim Kusz, Alicja Ratuszna

**Affiliations:** a Institute of Physics, University of Silesia, Uniwersytecka 4, 40-007 Katowice, Poland; b Institute of Materials Science, University of Silesia, Bankowa 12, 40-007 Katowice, Poland; c Institute of Chemistry, University of Silesia, Szkolna 9, 40-007 Katowice, Poland

## Abstract

In the title compound, C_48_H_38_N_4_O_8_·C_3_H_6_O_2_, the porphyrin mol­ecule is centrosymmetric. The propionic acid solvent mol­ecule is disordered over two sets of sites with equal occupancy factors. The porphyrin central core is almost planar, with an r.m.s. deviation of the fitted atoms of 0.045 Å. The substituent benzene rings make dihedral angles of 70.37 (4) and 66.95 (4)° with respect to the porphyrin core plane. The crystal structure is stabilized by an inter­esting network of hydrogen bonds. Porphyrin mol­ecules are connected by O—H⋯O hydrogen bonds creating ribbons running along the [101] direction. Weak C—H⋯O hydrogen bonds connect separate mol­ecular ribbons in the [110] direction, creating (-111) layers. Intra­molecular N—H⋯N hydrogen bonds also occur. The propionic acid molecules are connected by pairs of —H⋯O hydrogen bonds, creating dimers.

## Related literature

For the biological activity and potential applications of porphyrin mol­ecules, see: Allison *et al.* (2004 ▶); Dougherty *et al.* (1998 ▶); Agostinis *et al.* (2011 ▶); Szurko *et al.* (2009 ▶). For spectroscopic data, see Bonar-Law (1996 ▶).
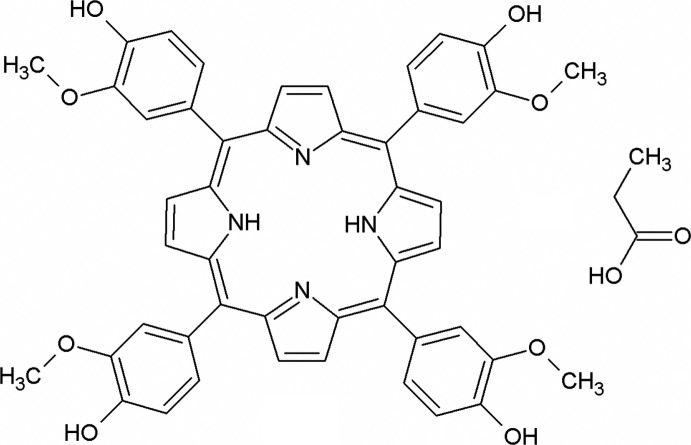


## Experimental

### 

#### Crystal data

C_48_H_38_N_4_O_8_·C_3_H_6_O_2_
*M* *_r_* = 872.90
Triclinic, 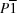

*a* = 6.8715 (5) Å
*b* = 12.0783 (7) Å
*c* = 14.3772 (10) Å
α = 112.850 (6)°
β = 98.560 (5)°
γ = 97.480 (5)°
*V* = 1063.97 (12) Å^3^
*Z* = 1
Cu *K*α radiation
μ = 0.78 mm^−1^
*T* = 100 K
0.10 × 0.03 × 0.02 mm

#### Data collection

Agilent SuperNova (Dual, Cu at zero, Atlas) diffractometer
Absorption correction: multi-scan (*CrysAlis PRO*; Agilent, 2011 ▶) *T*_min_ = 0.926, *T*_max_ = 0.985
9919 measured reflections
3688 independent reflections
3098 reflections with *I* > 2σ(*I*)
*R*_int_ = 0.028

#### Refinement

*R*[*F* ^2^ > 2σ(*F* ^2^)] = 0.038
*wR*(*F* ^2^) = 0.104
*S* = 1.03
3688 reflections
339 parameters
12 restraints
H atoms treated by a mixture of independent and constrained refinement
Δρ_max_ = 0.22 e Å^−3^
Δρ_min_ = −0.25 e Å^−3^

### 

Data collection: *CrysAlis PRO* (Agilent, 2011 ▶); cell refinement: *CrysAlis PRO*; data reduction: *CrysAlis PRO*; program(s) used to solve structure: *SHELXS97* (Sheldrick, 2008 ▶); program(s) used to refine structure: *SHELXL97* (Sheldrick, 2008 ▶); molecular graphics: *PLATON* (Spek, 2009 ▶) and *Mercury* (Macrae *et al.*, 2006 ▶); software used to prepare material for publication: *publCIF* (Westrip, 2010 ▶).

## Supplementary Material

Crystal structure: contains datablock(s) I, global. DOI: 10.1107/S1600536812036495/bt5999sup1.cif

Structure factors: contains datablock(s) I. DOI: 10.1107/S1600536812036495/bt5999Isup2.hkl

Additional supplementary materials:  crystallographic information; 3D view; checkCIF report

## Figures and Tables

**Table 1:** Hydrogen-bond geometry (Å, °)

*D*—H⋯*A*	*D*—H	H⋯*A*	*D*⋯*A*	*D*—H⋯*A*
N1—H1*N*⋯N2	0.85 (2)	2.392 (19)	2.9286 (19)	121.4 (15)
N1—H1*N*⋯N2^i^	0.85 (2)	2.377 (19)	2.9121 (19)	121.2 (15)
O1—H1*O*⋯O3	0.84 (2)	2.17 (2)	2.6655 (17)	117.4 (19)
O2—H2*O*⋯O4	0.90 (2)	2.18 (2)	2.6726 (16)	113.8 (18)
O2—H2*O*⋯O1^ii^	0.90 (2)	2.03 (2)	2.8588 (17)	151 (2)
C10—H10⋯O3^iii^	0.98 (2)	2.46 (2)	3.4085 (19)	162.0 (16)
C23—H23*B*⋯O3^ii^	0.99 (3)	2.51 (3)	3.383 (2)	147.3 (19)
C23—H23*C*⋯O5^ii^	1.00 (3)	2.43 (3)	3.189 (5)	132.3 (19)
O5—H5*A*⋯O6^ii^	0.84	1.77	2.608 (9)	173

Symmetry codes: (i) 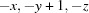; (ii) 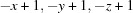; (iii) 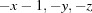.

**Fig. 1.**
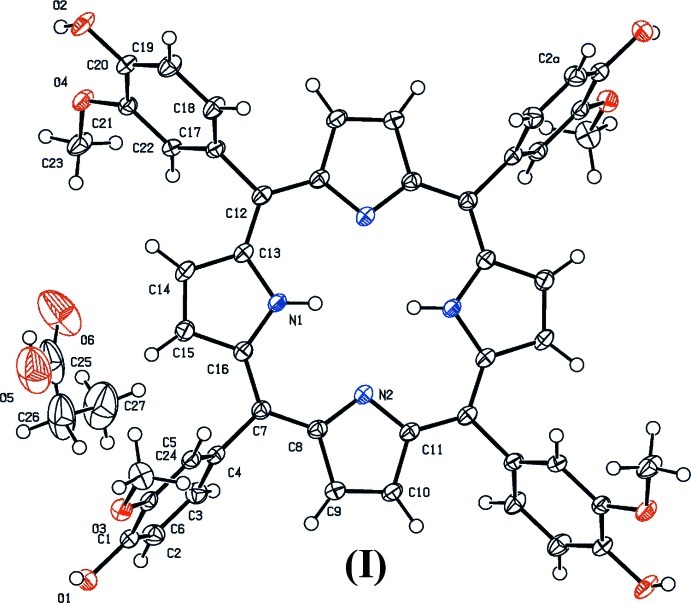
The molecular structure of compound (I), showing atom-labelling scheme. The molecule is centrosymmetric and only the unique atoms of the asymmetric unit are labelled. Ellipsoids representing displacement parameters are drown at the 50% probability level.

**Fig. 2.**
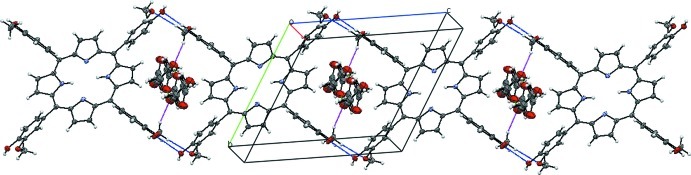
Scheme of network of hydrogen bonds in (I). O2—H2O···O1ii [symmetry codes: (ii) 1 - x,1 - y,1 - z] hydrogen bonds are marked by blue lines connecting separated molecules into molecular ribbons. Magenta lines indicate C23—H23B···O3ii and C23—H23C···O5ii hydrogen bonds and green lines indicate O5—H5A···O6ii hydrogen bond connecting propionic acid molecules.

## supplementary crystallographic information

### Comment

Photodynamic therapy (PDT) for cancer treatment is still developed method. PDT process is result of the photochemistry reactions, where the photosensitizers are activated by the light and during the deactivation their energy is used to excite molecular O_2_ to the singlet state ^1^O_2_*. The excited oxygen as well as the other species (ROS) are highly toxic and oxidize organic substrates found within tumour cells, leading to its destruction. Effectiveness of this technique is determined by the properties exhibited by the photosensitizers. For this reason we try to obtain a well defined compounds, which should have among the others: chemical purity, stability, good solubility in water or fat, lack of aggregation, optimal quantum yield of fluorescence and long lifetime of triplet states [Allison *et al.*, 2004; Dougherty *et al.*, 1998; Agostinis *et al.*, 2011; Szurko *et al.*, 2009]. Promising compounds for application in PDT are the porhyrins, due to their photosensibilization properties.

This paper presents the crystal structure of *meso*-tetra(4-hydroxy-3-methoxyphenyl)porphyrin (I), which is good a candidate as a starting material for synthesis of a new potential anticancer photosensitizer. Compound (I) crystallizes in triclinic system with one porphyrin molecule in the unit cell. Crystal structure contains also one propionic acid solvent molecule per one porphyrin molecule (Fig.1). The solvent molecule is disordered and can occupy two positions in the unit cell with equal occupancy factors. Porphyrin molecule is centrosymmetric with two sets of benzene rings orientations. Central core of porphyrin molecule is approximately planar with r.m.s. deviation of fitted atoms equal to 0.045 Å. The largest distance from one atom (N1) to the average plane is 0.1057 (13) Å. The angles of substituent benzene rings with the porphyrin core plane are 70.37 (4)° and 66.95 (4)°. Porphyrin molecules occupy (596) planes creating π-π stacking structure. The distance between centroids of two pyrrolide ring and pyrrole rings is 4.232 Å. Two sets of methylene groups are almost coplanar with benzene rings with largest distance to average plane equal to 0.0982 (34) Å and 0.1053 (31) Å for atoms C23 and C24 respectively. The two torsion angles are as follows: C5—C6—O3—C24 = -4.5 (2)° and C22—C21—O4—C23 = 4.4 (2)°.

Propionic acid molecules are connected by two O5—H5A···O6^ii^ [symmetry codes: (ii) 1 - *x*,1 - *y*,1 - *z*] hydrogen bonds creating dimmers along [100] direction. Porphyrin molecules are connected by O2—H2O···O1^ii^ hydrogen bonds creating ribbons running along [101] directions. Weak C10—H10···O3^iii^ [symmetry codes: (iii) -1 - *x*, -*y*,-*z*] hydrogen bonds connect separated molecular ribbons in [110] direction creating (111) layers. Ribbons of porphyrin molecules are intersecting with direction of propionic acid molecules dimmers and additional C23—H23B···O3^ii^ and C23—H23C···O5^ii^ hydrogen bonds are created (Fig.2). π-π stacking and interactions with propionic acid molecules stabilize the crystal structure of presented compound. Two types of intramolecular hydrogen bonds O1—H1O···O3 and O2—H2O···O4 are present connecting methylene group and oxygen atom connected to benzene rings. Detailed information regarding hydrogen bonds in the compound are stated in Table 1.

### Experimental

Chemicals and solvents were purchased from commercial sources and used as received. Synthesis of *meso*-tetra(4-hydroxy-3-methoxyphenyl)porphyrin (I) was performed as fallows. 8.8 g (0.072 m) of vanillin was added into 300 ml of propionic acid. The mixture was boiled until the all aldehyde was dissolved. After this 5 ml (0.072 m) of pyrrole was added and the solution was boiled for 1.5 h. Then about 200 ml of propionic acid was distilled off, the residue was cooled to ambient temperature and neutralized with saturated solution of NaHCO_3_. The precipitate was filtered and washed with chloroform until the filtrate was colourless. The product of the reaction was purified by column chromatography (silica gel/chloroform:ethyl acetate).

The single crystals of (I) were obtained directly from precipitate after reaction procedure (before column chromatography purification).

All spectroscopic data were in accordance with literature [Bonar-Law, 1996].

### Refinement

Non-hydrogen atoms were refined with anisotropic displacement parameters. The aromatic, methyl and hydroxyl hydrogen atoms were treated as "riding" on their parent carbon atoms with C—H = 0.96 Å, C—H = 0.98 Å and C—H = 0.84 Å respectively. Atomic displacement parameters of hydrogen atoms equal to 1.2 times the value of the equivalent atomic displacement parameters of the parent carbon atom (*U*_iso_(H) = 1.2*U*_eq_(C)) for aromatic hydrogen atoms and 1.5 times the value of the equivalent atomic displacement parameters of the parent carbon atom (*U*_iso_(H) = 1.5*U*_eq_(C)) for methyl and hydroxyl hydrogen atoms. Hydrogen atoms, which take part in hydrogen bonding, were located in a difference Fourier map (*ΔF*) and they were refined freely with isotropic displacement parameters. Similar-ADP restraint (SIMU) was applied to carbon atoms (C25, C26 and C27) in disordered propionic acid molecule.

### Crystal data

**Table:**

C_48_H_38_N_4_O_8_·C_3_H_6_O_2_	*Z* = 1
*M**_r_* = 872.90	*F*(000) = 458
Triclinic, *P*1	*D*_x_ = 1.362 Mg m^−^^3^
Hall symbol: -P 1	Cu *K*α radiation, λ = 1.54184 Å
*a* = 6.8715 (5) Å	Cell parameters from 4163 reflections
*b* = 12.0783 (7) Å	θ = 3.4–65.9°
*c* = 14.3772 (10) Å	µ = 0.78 mm^−^^1^
α = 112.850 (6)°	*T* = 100 K
β = 98.560 (5)°	Polyhedron, black
γ = 97.480 (5)°	0.10 × 0.03 × 0.02 mm
*V* = 1063.97 (12) Å^3^	

### Data collection

**Table:**

Agilent SuperNova (Dual, Cu at zero, Atlas) diffractometer	3688 independent reflections
Radiation source: SuperNova (Cu) X-ray Source	3098 reflections with *I* > 2σ(*I*)
Mirror monochromator	*R*_int_ = 0.028
Detector resolution: 10.4498 pixels mm^-1^	θ_max_ = 66.0°, θ_min_ = 3.4°
ω scans	*h* = −8→8
Absorption correction: multi-scan (*CrysAlis PRO*; Agilent, 2011)	*k* = −13→14
*T*_min_ = 0.926, *T*_max_ = 0.985	*l* = −15→17
9919 measured reflections	

### Refinement

**Table:**

Refinement on *F*^2^	Secondary atom site location: difference Fourier map
Least-squares matrix: full	Hydrogen site location: inferred from neighbouring sites
*R*[*F*^2^ > 2σ(*F*^2^)] = 0.038	H atoms treated by a mixture of independent and constrained refinement
*wR*(*F*^2^) = 0.104	*w* = 1/[σ^2^(*F*_o_^2^) + (0.0536*P*)^2^ + 0.3935*P*] where *P* = (*F*_o_^2^ + 2*F*_c_^2^)/3
*S* = 1.03	(Δ/σ)_max_ < 0.001
3688 reflections	Δρ_max_ = 0.22 e Å^−^^3^
339 parameters	Δρ_min_ = −0.25 e Å^−^^3^
12 restraints	Extinction correction: *SHELXL97* (Sheldrick, 2008), Fc^*^=kFc[1+0.001xFc^2^λ^3^/sin(2θ)]^-1/4^
Primary atom site location: structure-invariant direct methods	Extinction coefficient: 0.0040 (5)

### Special details

**Table:**

Experimental. Absorption correction: CrysAlisPro, Agilent Technologies, Version 1.171.35.19 Empirical absorption correction using spherical harmonics, implemented in SCALE3 ABSPACK scaling algorithm.
Geometry. All e.s.d.'s (except the e.s.d. in the dihedral angle between two l.s. planes) are estimated using the full covariance matrix. The cell e.s.d.'s are taken into account individually in the estimation of e.s.d.'s in distances, angles and torsion angles; correlations between e.s.d.'s in cell parameters are only used when they are defined by crystal symmetry. An approximate (isotropic) treatment of cell e.s.d.'s is used for estimating e.s.d.'s involving l.s. planes.
Refinement. Refinement of *F*^2^ against ALL reflections. The weighted *R*-factor *wR* and goodness of fit *S* are based on *F*^2^, conventional *R*-factors *R* are based on *F*, with *F* set to zero for negative *F*^2^. The threshold expression of *F*^2^ > σ(*F*^2^) is used only for calculating *R*-factors(gt) *etc*. and is not relevant to the choice of reflections for refinement. *R*-factors based on *F*^2^ are statistically about twice as large as those based on *F*, and *R*- factors based on ALL data will be even larger.

### Fractional atomic coordinates and isotropic or equivalent isotropic displacement parameters (Å^2^)

**Table:**

	*x*	*y*	*z*	*U*_iso_*/*U*_eq_	Occ. (<1)
O1	−0.28230 (18)	0.01112 (11)	0.33140 (9)	0.0254 (3)	
H1O	−0.211 (3)	−0.042 (2)	0.3180 (17)	0.038*	
O2	1.21357 (17)	0.83439 (12)	0.45275 (9)	0.0294 (3)	
H2O	1.190 (3)	0.882 (2)	0.5143 (19)	0.044*	
O3	−0.00783 (17)	−0.03851 (10)	0.21636 (8)	0.0237 (3)	
O4	0.87195 (16)	0.87779 (11)	0.51416 (8)	0.0256 (3)	
N1	0.1653 (2)	0.46061 (12)	0.11394 (10)	0.0188 (3)	
H1N	0.101 (3)	0.4735 (17)	0.0650 (15)	0.023*	
N2	−0.24395 (19)	0.37519 (11)	−0.01361 (10)	0.0186 (3)	
C1	−0.2385 (2)	0.08075 (14)	0.27810 (12)	0.0216 (4)	
C2	−0.3381 (3)	0.17440 (15)	0.28535 (13)	0.0256 (4)	
H2	−0.4365	0.1896	0.3262	0.031*	
C3	−0.2950 (2)	0.24694 (15)	0.23292 (13)	0.0241 (4)	
H3	−0.3647	0.3114	0.2381	0.029*	
C4	−0.1513 (2)	0.22622 (14)	0.17301 (12)	0.0198 (3)	
C5	−0.0510 (2)	0.13016 (14)	0.16546 (12)	0.0205 (3)	
H5	0.0473	0.1147	0.1246	0.025*	
C6	−0.0946 (2)	0.05773 (14)	0.21729 (12)	0.0201 (3)	
C7	−0.1047 (2)	0.30658 (14)	0.11882 (11)	0.0188 (3)	
C8	−0.2574 (2)	0.30429 (14)	0.04101 (12)	0.0194 (3)	
C9	−0.4521 (2)	0.22058 (15)	0.00422 (12)	0.0228 (4)	
H9	−0.4984	0.1625	0.0297	0.027*	
C10	−0.5542 (2)	0.24117 (15)	−0.07284 (12)	0.0224 (4)	
H10	−0.689 (3)	0.1991 (18)	−0.1155 (15)	0.027*	
C11	−0.4238 (2)	0.33809 (14)	−0.08348 (12)	0.0193 (3)	
C12	0.4759 (2)	0.61748 (14)	0.15889 (12)	0.0188 (3)	
C13	0.3540 (2)	0.52417 (14)	0.17144 (12)	0.0189 (3)	
C14	0.3994 (2)	0.48061 (14)	0.24959 (12)	0.0225 (4)	
H14	0.5208	0.5076	0.3007	0.027*	
C15	0.2393 (2)	0.39374 (14)	0.23837 (12)	0.0228 (4)	
H15	0.2294	0.3494	0.2800	0.027*	
C16	0.0885 (2)	0.38112 (14)	0.15268 (12)	0.0195 (3)	
C17	0.6721 (2)	0.67684 (14)	0.23575 (12)	0.0191 (3)	
C18	0.8540 (2)	0.65879 (15)	0.20740 (13)	0.0247 (4)	
H18	0.8553	0.6091	0.1377	0.030*	
C19	1.0343 (2)	0.71304 (15)	0.28061 (13)	0.0260 (4)	
H19	1.1579	0.7003	0.2604	0.031*	
C20	1.0351 (2)	0.78502 (14)	0.38213 (12)	0.0217 (3)	
C21	0.8527 (2)	0.80458 (14)	0.41172 (12)	0.0194 (3)	
C22	0.6726 (2)	0.75083 (14)	0.33878 (12)	0.0192 (3)	
H22	0.5490	0.7643	0.3589	0.023*	
C23	0.6888 (3)	0.8927 (2)	0.54936 (14)	0.0344 (5)	
H23A	0.600 (4)	0.933 (2)	0.5120 (19)	0.052*	
H23B	0.729 (4)	0.944 (2)	0.624 (2)	0.052*	
H23C	0.608 (4)	0.811 (2)	0.5363 (19)	0.052*	
C24	0.1311 (3)	−0.07391 (17)	0.15062 (14)	0.0321 (4)	
H24A	0.2460	−0.0054	0.1724	0.048*	
H24B	0.0641	−0.0956	0.0789	0.048*	
H24C	0.1785	−0.1450	0.1556	0.048*	
O6	0.3201 (9)	0.5783 (3)	0.5140 (3)	0.1001 (17)	0.50
O5	0.2982 (10)	0.3840 (4)	0.4875 (3)	0.0992 (17)	0.50
H5A	0.4195	0.4002	0.4851	0.149*	0.50
C25	0.2215 (14)	0.4812 (6)	0.5036 (4)	0.078 (2)	0.50
C26	0.0013 (18)	0.4670 (7)	0.5103 (7)	0.086 (3)	0.50
H26A	−0.0735	0.3851	0.4583	0.103*	0.50
H26B	−0.0093	0.4716	0.5796	0.103*	0.50
C27	−0.0933 (19)	0.5642 (10)	0.4919 (9)	0.099 (4)	0.50
H27A	−0.2359	0.5497	0.4941	0.148*	0.50
H27B	−0.0808	0.5611	0.4240	0.148*	0.50
H27C	−0.0250	0.6452	0.5458	0.148*	0.50

### Atomic displacement parameters (Å^2^)

**Table:**

	*U*^11^	*U*^22^	*U*^33^	*U*^12^	*U*^13^	*U*^23^
O1	0.0275 (6)	0.0259 (6)	0.0237 (6)	0.0023 (5)	0.0049 (5)	0.0125 (5)
O2	0.0141 (6)	0.0390 (7)	0.0221 (6)	0.0023 (5)	−0.0026 (5)	0.0022 (5)
O3	0.0252 (6)	0.0214 (6)	0.0243 (6)	0.0060 (5)	0.0046 (5)	0.0092 (5)
O4	0.0159 (6)	0.0363 (7)	0.0161 (5)	0.0004 (5)	0.0021 (4)	0.0039 (5)
N1	0.0152 (6)	0.0196 (7)	0.0180 (7)	0.0012 (5)	−0.0007 (5)	0.0065 (5)
N2	0.0166 (6)	0.0187 (6)	0.0171 (6)	0.0030 (5)	0.0012 (5)	0.0051 (5)
C1	0.0211 (8)	0.0212 (8)	0.0180 (8)	−0.0023 (6)	−0.0003 (6)	0.0070 (6)
C2	0.0223 (8)	0.0285 (9)	0.0253 (8)	0.0037 (7)	0.0071 (7)	0.0102 (7)
C3	0.0226 (8)	0.0243 (8)	0.0260 (8)	0.0060 (7)	0.0053 (7)	0.0107 (7)
C4	0.0176 (8)	0.0195 (8)	0.0172 (7)	−0.0008 (6)	−0.0016 (6)	0.0055 (6)
C5	0.0177 (8)	0.0214 (8)	0.0176 (7)	0.0004 (6)	0.0010 (6)	0.0051 (6)
C6	0.0191 (8)	0.0179 (7)	0.0171 (7)	0.0001 (6)	−0.0024 (6)	0.0043 (6)
C7	0.0181 (8)	0.0184 (7)	0.0172 (7)	0.0026 (6)	0.0030 (6)	0.0053 (6)
C8	0.0188 (8)	0.0179 (7)	0.0178 (7)	0.0017 (6)	0.0032 (6)	0.0044 (6)
C9	0.0203 (8)	0.0226 (8)	0.0221 (8)	−0.0012 (6)	0.0018 (6)	0.0086 (7)
C10	0.0168 (8)	0.0235 (8)	0.0212 (8)	−0.0011 (6)	−0.0001 (6)	0.0064 (7)
C11	0.0163 (7)	0.0186 (8)	0.0176 (7)	0.0018 (6)	0.0020 (6)	0.0032 (6)
C12	0.0158 (8)	0.0180 (7)	0.0176 (7)	0.0035 (6)	0.0019 (6)	0.0028 (6)
C13	0.0161 (7)	0.0179 (7)	0.0178 (7)	0.0029 (6)	0.0012 (6)	0.0035 (6)
C14	0.0177 (8)	0.0216 (8)	0.0219 (8)	0.0017 (6)	−0.0038 (6)	0.0062 (7)
C15	0.0225 (8)	0.0218 (8)	0.0220 (8)	0.0028 (6)	−0.0001 (6)	0.0094 (7)
C16	0.0190 (8)	0.0183 (7)	0.0185 (7)	0.0033 (6)	0.0023 (6)	0.0057 (6)
C17	0.0164 (8)	0.0169 (7)	0.0208 (8)	0.0005 (6)	0.0001 (6)	0.0069 (6)
C18	0.0195 (8)	0.0273 (9)	0.0192 (8)	0.0046 (7)	0.0017 (6)	0.0023 (7)
C19	0.0167 (8)	0.0298 (9)	0.0255 (9)	0.0061 (7)	0.0038 (7)	0.0050 (7)
C20	0.0150 (8)	0.0233 (8)	0.0220 (8)	0.0008 (6)	−0.0013 (6)	0.0071 (7)
C21	0.0183 (8)	0.0199 (8)	0.0170 (7)	0.0011 (6)	0.0023 (6)	0.0059 (6)
C22	0.0145 (7)	0.0209 (8)	0.0203 (8)	0.0010 (6)	0.0018 (6)	0.0081 (6)
C23	0.0182 (9)	0.0506 (12)	0.0210 (9)	0.0000 (8)	0.0060 (7)	0.0027 (8)
C24	0.0372 (10)	0.0318 (9)	0.0324 (10)	0.0161 (8)	0.0136 (8)	0.0138 (8)
O6	0.192 (5)	0.039 (2)	0.067 (3)	0.009 (3)	0.050 (3)	0.0162 (18)
O5	0.165 (5)	0.054 (2)	0.073 (3)	0.006 (3)	−0.001 (3)	0.036 (2)
C25	0.157 (7)	0.038 (3)	0.025 (2)	0.001 (4)	−0.001 (3)	0.011 (2)
C26	0.153 (8)	0.037 (5)	0.050 (3)	0.002 (5)	−0.017 (5)	0.020 (3)
C27	0.138 (11)	0.053 (6)	0.092 (6)	0.010 (5)	−0.026 (6)	0.037 (4)

### Geometric parameters (Å, º)

**Table:**

O1—C1	1.3742 (19)	C12—C11^i^	1.406 (2)
O1—H1O	0.84 (2)	C12—C17	1.497 (2)
O2—C20	1.3637 (19)	C13—C14	1.426 (2)
O2—H2O	0.90 (2)	C14—C15	1.361 (2)
O3—C6	1.3704 (19)	C14—H14	0.9500
O3—C24	1.431 (2)	C15—C16	1.431 (2)
O4—C21	1.3670 (19)	C15—H15	0.9500
O4—C23	1.432 (2)	C17—C18	1.389 (2)
N1—C16	1.372 (2)	C17—C22	1.401 (2)
N1—C13	1.372 (2)	C18—C19	1.392 (2)
N1—H1N	0.85 (2)	C18—H18	0.9500
N2—C11	1.369 (2)	C19—C20	1.377 (2)
N2—C8	1.372 (2)	C19—H19	0.9500
C1—C2	1.375 (2)	C20—C21	1.402 (2)
C1—C6	1.400 (2)	C21—C22	1.389 (2)
C2—C3	1.391 (2)	C22—H22	0.9500
C2—H2	0.9500	C23—H23A	1.04 (3)
C3—C4	1.389 (2)	C23—H23B	0.99 (3)
C3—H3	0.9500	C23—H23C	1.00 (3)
C4—C5	1.402 (2)	C24—H24A	0.9800
C4—C7	1.494 (2)	C24—H24B	0.9800
C5—C6	1.384 (2)	C24—H24C	0.9800
C5—H5	0.9500	O6—C25	1.218 (8)
C7—C16	1.402 (2)	O5—C25	1.303 (9)
C7—C8	1.405 (2)	O5—H5A	0.8400
C8—C9	1.454 (2)	C25—C26	1.522 (16)
C9—C10	1.345 (2)	C26—C27	1.503 (13)
C9—H9	0.9500	C26—H26A	0.9900
C10—C11	1.450 (2)	C26—H26B	0.9900
C10—H10	0.98 (2)	C27—H27A	0.9800
C11—C12^i^	1.406 (2)	C27—H27B	0.9800
C12—C13	1.401 (2)	C27—H27C	0.9800
			
C1—O1—H1O	107.0 (15)	C14—C15—H15	126.1
C20—O2—H2O	108.6 (15)	C16—C15—H15	126.1
C6—O3—C24	117.77 (13)	N1—C16—C7	126.76 (14)
C21—O4—C23	116.35 (12)	N1—C16—C15	106.89 (13)
C16—N1—C13	110.09 (13)	C7—C16—C15	126.26 (14)
C16—N1—H1N	124.7 (13)	C18—C17—C22	119.17 (14)
C13—N1—H1N	124.9 (13)	C18—C17—C12	121.39 (14)
C11—N2—C8	105.46 (12)	C22—C17—C12	119.44 (14)
O1—C1—C2	118.99 (15)	C17—C18—C19	120.38 (15)
O1—C1—C6	121.02 (15)	C17—C18—H18	119.8
C2—C1—C6	119.99 (15)	C19—C18—H18	119.8
C1—C2—C3	120.07 (15)	C20—C19—C18	120.56 (15)
C1—C2—H2	120.0	C20—C19—H19	119.7
C3—C2—H2	120.0	C18—C19—H19	119.7
C4—C3—C2	120.75 (15)	O2—C20—C19	119.30 (15)
C4—C3—H3	119.6	O2—C20—C21	121.01 (14)
C2—C3—H3	119.6	C19—C20—C21	119.68 (14)
C3—C4—C5	118.91 (15)	O4—C21—C22	125.60 (14)
C3—C4—C7	119.91 (14)	O4—C21—C20	114.51 (13)
C5—C4—C7	121.18 (14)	C22—C21—C20	119.88 (14)
C6—C5—C4	120.30 (15)	C21—C22—C17	120.32 (15)
C6—C5—H5	119.9	C21—C22—H22	119.8
C4—C5—H5	119.9	C17—C22—H22	119.8
O3—C6—C5	126.06 (14)	O4—C23—H23A	112.4 (14)
O3—C6—C1	113.96 (13)	O4—C23—H23B	105.9 (14)
C5—C6—C1	119.98 (15)	H23A—C23—H23B	111.4 (19)
C16—C7—C8	125.43 (14)	O4—C23—H23C	109.9 (14)
C16—C7—C4	116.49 (14)	H23A—C23—H23C	108 (2)
C8—C7—C4	118.05 (13)	H23B—C23—H23C	109 (2)
N2—C8—C7	125.96 (14)	O3—C24—H24A	109.5
N2—C8—C9	110.38 (13)	O3—C24—H24B	109.5
C7—C8—C9	123.62 (14)	H24A—C24—H24B	109.5
C10—C9—C8	106.76 (14)	O3—C24—H24C	109.5
C10—C9—H9	126.6	H24A—C24—H24C	109.5
C8—C9—H9	126.6	H24B—C24—H24C	109.5
C9—C10—C11	106.70 (14)	C25—O5—H5A	109.5
C9—C10—H10	128.0 (11)	O6—C25—O5	122.2 (9)
C11—C10—H10	125.3 (11)	O6—C25—C26	121.8 (7)
N2—C11—C12^i^	125.73 (14)	O5—C25—C26	116.0 (7)
N2—C11—C10	110.70 (14)	C27—C26—C25	112.1 (6)
C12^i^—C11—C10	123.52 (14)	C27—C26—H26A	109.2
C13—C12—C11^i^	125.08 (14)	C25—C26—H26A	109.2
C13—C12—C17	116.23 (14)	C27—C26—H26B	109.2
C11^i^—C12—C17	118.62 (13)	C25—C26—H26B	109.2
N1—C13—C12	126.83 (14)	H26A—C26—H26B	107.9
N1—C13—C14	106.81 (13)	C26—C27—H27A	109.5
C12—C13—C14	126.31 (14)	C26—C27—H27B	109.5
C15—C14—C13	108.34 (14)	H27A—C27—H27B	109.5
C15—C14—H14	125.8	C26—C27—H27C	109.5
C13—C14—H14	125.8	H27A—C27—H27C	109.5
C14—C15—C16	107.86 (14)	H27B—C27—H27C	109.5
			
O1—C1—C2—C3	179.38 (14)	C17—C12—C13—N1	176.90 (14)
C6—C1—C2—C3	−0.5 (2)	C11^i^—C12—C13—C14	−177.37 (15)
C1—C2—C3—C4	−0.1 (2)	C17—C12—C13—C14	−0.3 (2)
C2—C3—C4—C5	0.5 (2)	N1—C13—C14—C15	−0.45 (18)
C2—C3—C4—C7	−178.92 (14)	C12—C13—C14—C15	177.19 (15)
C3—C4—C5—C6	−0.2 (2)	C13—C14—C15—C16	−0.24 (18)
C7—C4—C5—C6	179.19 (14)	C13—N1—C16—C7	175.63 (15)
C24—O3—C6—C5	−4.5 (2)	C13—N1—C16—C15	−1.14 (17)
C24—O3—C6—C1	175.86 (14)	C8—C7—C16—N1	−2.8 (3)
C4—C5—C6—O3	−179.98 (13)	C4—C7—C16—N1	179.17 (14)
C4—C5—C6—C1	−0.4 (2)	C8—C7—C16—C15	173.42 (15)
O1—C1—C6—O3	0.5 (2)	C4—C7—C16—C15	−4.7 (2)
C2—C1—C6—O3	−179.61 (14)	C14—C15—C16—N1	0.84 (18)
O1—C1—C6—C5	−179.11 (13)	C14—C15—C16—C7	−175.96 (15)
C2—C1—C6—C5	0.8 (2)	C13—C12—C17—C18	110.22 (18)
C3—C4—C7—C16	113.71 (17)	C11^i^—C12—C17—C18	−72.5 (2)
C5—C4—C7—C16	−65.69 (19)	C13—C12—C17—C22	−68.89 (19)
C3—C4—C7—C8	−64.5 (2)	C11^i^—C12—C17—C22	108.39 (17)
C5—C4—C7—C8	116.09 (16)	C22—C17—C18—C19	0.3 (2)
C11—N2—C8—C7	177.38 (15)	C12—C17—C18—C19	−178.78 (15)
C11—N2—C8—C9	−0.18 (17)	C17—C18—C19—C20	0.2 (3)
C16—C7—C8—N2	−0.8 (3)	C18—C19—C20—O2	178.29 (16)
C4—C7—C8—N2	177.23 (14)	C18—C19—C20—C21	−0.5 (3)
C16—C7—C8—C9	176.43 (15)	C23—O4—C21—C22	4.4 (2)
C4—C7—C8—C9	−5.5 (2)	C23—O4—C21—C20	−175.46 (16)
N2—C8—C9—C10	0.38 (18)	O2—C20—C21—O4	1.4 (2)
C7—C8—C9—C10	−177.25 (15)	C19—C20—C21—O4	−179.76 (15)
C8—C9—C10—C11	−0.40 (18)	O2—C20—C21—C22	−178.45 (15)
C8—N2—C11—C12^i^	−177.59 (15)	C19—C20—C21—C22	0.4 (2)
C8—N2—C11—C10	−0.07 (17)	O4—C21—C22—C17	−179.70 (14)
C9—C10—C11—N2	0.31 (18)	C20—C21—C22—C17	0.2 (2)
C9—C10—C11—C12^i^	177.89 (15)	C18—C17—C22—C21	−0.5 (2)
C16—N1—C13—C12	−176.62 (15)	C12—C17—C22—C21	178.62 (14)
C16—N1—C13—C14	1.00 (17)	O6—C25—C26—C27	20.7 (8)
C11^i^—C12—C13—N1	−0.2 (3)	O5—C25—C26—C27	−160.2 (5)

Symmetry code: (i) −*x*, −*y*+1, −*z*.

### Hydrogen-bond geometry (Å, º)

**Table:**

*D*—H···*A*	*D*—H	H···*A*	*D*···*A*	*D*—H···*A*
N1—H1*N*···N2	0.85 (2)	2.392 (19)	2.9286 (19)	121.4 (15)
N1—H1*N*···N2^i^	0.85 (2)	2.377 (19)	2.9121 (19)	121.2 (15)
O1—H1*O*···O3	0.84 (2)	2.17 (2)	2.6655 (17)	117.4 (19)
O2—H2*O*···O4	0.90 (2)	2.18 (2)	2.6726 (16)	113.8 (18)
O2—H2*O*···O1^ii^	0.90 (2)	2.03 (2)	2.8588 (17)	151 (2)
C10—H10···O3^iii^	0.98 (2)	2.46 (2)	3.4085 (19)	162.0 (16)
C23—H23*B*···O3^ii^	0.99 (3)	2.51 (3)	3.383 (2)	147.3 (19)
C23—H23*C*···O5^ii^	1.00 (3)	2.43 (3)	3.189 (5)	132.3 (19)
O5—H5*A*···O6^ii^	0.84	1.77	2.608 (9)	173

Symmetry codes: (i) −*x*, −*y*+1, −*z*; (ii) −*x*+1, −*y*+1, −*z*+1; (iii) −*x*−1, −*y*, −*z*.
